# The Journey to a FAIR CORE DATA SET for Diabetes Research in Germany

**DOI:** 10.1038/s41597-024-03882-0

**Published:** 2024-10-21

**Authors:** Esther Thea Inau, Angela Dedié, Ivona Anastasova, Renate Schick, Yaroslav Zdravomyslov, Brigitte Fröhlich, Andreas L. Birkenfeld, Martin Hrabě de Angelis, Michael Roden, Atinkut Alamirrew Zeleke, Martin Preusse, Dagmar Waltemath

**Affiliations:** 1 Medical Informatics Laboratory, University Medicine Greifswald, Greifswald, Germany; 2 German Center for Diabetes Research (DZD), München-Neuherberg, Germany; 3 German Center for Diabetes Research (DZD), Tübingen, Germany; 4 Institute for Diabetes Research and Metabolic Diseases of the Helmholtz Zentrum München at the University of Tübingen (IDM), Tübingen, Germany; 5 Department of Diabetology, Endocrinology, and Nephrology, University Clinic Tübingen, Eberhard Karls University Tübingen, Tübingen, Germany; 6 Institute of Experimental Genetics and German Mouse Clinic, Helmholtz Munich, Neuherberg, Germany; 7 Chair of Experimental Genetics, TUM School of Life Sciences (SoLS), Technische Universität München, Freising, Germany; 8 German Center for Diabetes Research (DZD), Düsseldorf, Germany; 9 Department of Endocrinology and Diabetology, Medical Faculty and University Hospital Düsseldorf, Heinrich-Heine-University Düsseldorf, Düsseldorf, Germany; 10 Institute for Clinical Diabetology, German Diabetes Center, Leibniz Center for Diabetes Research at Heinrich-Heine-University Düsseldorf, Düsseldorf, Germany

**Keywords:** Endocrine system and metabolic diseases, Research data

## Abstract

The German Center for Diabetes Research (DZD) established a core data set (CDS) of clinical parameters relevant for diabetes research in 2021. The CDS is central to the design of current and future DZD studies. Here, we describe the process and outcomes of FAIRifying the initial version of the CDS. We first did a baseline evaluation of the FAIRness using the FAIR Data Maturity Model. The FAIRification process and the results of this assessment led us to convert the CDS into the recommended format for spreadsheets, annotating the parameters with standardized medical codes, licensing the data set, enriching the data set with metadata, and indexing the metadata. The FAIRified version of the CDS is more suitable for data sharing in diabetes research across DZD sites and beyond. It contributes to the reusability of health research studies.

## Introduction

The German Center for Diabetes Research (*Deutsches Zentrum für Diabetesforschung *- DZD) conducts large clinical multicenter studies in the field of diabetes and metabolic research^1^. It is part of the German Centers for Health Research (Deutsche Zentrender Gesundheitsforschung - DZG) which focus on novel therapies for diabetes, infections, lung diseases, cancer, mental disorders, cardiovascular and neurodegenerative diseases^2–7^. In this vein, a core data set (CDS) provides the descriptions of variables and definitions that are relevant for clinical research in an information database for purposes of consistency, data validity and reliability^8,9^. Analysis of clinical data integrated from multiple sources has been used to generate critical information that supports clinical research^10,11^. Data exchange among different levels of healthcare is also linked to better health service management and improved care for persons with diseases^12^. However, the use of heterogeneous systems to collect different types of data, typically maintained in various formats, impedes both data exchange and consolidative data analysis for research^10,12^.

A CDS is typically presented to the audience in human-readable format to help the end-user properly interpret the meaning of the associated data^13^. Machine-readable formats are designed to allow computers to easily process the data, which requires the data to be structured in a specific and standardized way^14^. Machine-readable formats also support data encoding and exchange between heterogeneous systems to facilitate reporting and standard queries^15^. The development of a CDS in various scientific spheres has shown to be a valuable component when sharing or integrating complex data from multiple data sources across different systems^16^. The design of a CDS enables harmonization and standardization in the collection, measurement and reporting of minimal information necessary for collaborative research^17^. For example, the CDS for the German Medical Informatics Initiative and the NFDI4Health Metadata Schema both employ a standardized framework to capture essential information, promote consistency, efficiency and comparability in data management and analysis^18,19^.

In 2021 the DZD established a CDS containing a list of clinical parameters relevant for joint studies in diabetes research^20^. The DZD CORE DATA SET (DZD CDS) is designed as the core component of clinical studies conducted by the DZD. A detailed description of each parameter contributes to standardization of data collection and ensures that the data is uniformly designated, defined, and recorded in the same format across studies. The first version of the DZD CDS was published for internal use on the DZD website and has since become a mandatory component for the design of all new DZD clinical studies^20,21^. To date, the DZD CDS has already been implemented in various DZD studies such as the Influence of intermittent fasting on insulin secretion (IFIS), ClinicalTrials.gov ID: NCT04607096 and the SGLT2 Inhibition in Addition to Lifestyle Intervention and Risk for Complications in Subtypes of Patients With Prediabetes (LIFETIME), ClinicalTrials.gov ID: NCT06054035^22,23^. It serves to contribute to the harmonization of data for diabetes research in general, thus the need to enhance its sustainability, reproducibility and shareability. Work is underway to establish a common CDS across the German Centers for Health Research (DZG). The parameters of this overarching core data set are published in the Medical Data Models (MDM) portal and will be part of the next version of the DZD CDS^24^.

The implementation of a formalised, provenance-enabled and semantically enriched representation of (meta)data leads to more findable, accessible, interoperable and reusable (FAIR) data^25^. The metadata adds value to data and saves time spent on data exploration, data selection during access and data processing which is key to realising the full capabilities of research^25,26^. The primary goal of this work was to enhance the value of the DZD CORE DATA SET (DZD CDS) through a two-fold approach: first, by conducting a baseline FAIR assessment, followed by implementing targeted FAIRification measures. As a result, a FAIRer version of the DZD CDS has been developed which is the main success benchmark of this work. The FAIRification process was imperative for both the data proprietors and diabetes research community, as it facilitates improved data management and sharing capabilities.

## Methods

We started our FAIRification journey by conducting a baseline assessment of the following FAIR aspects:

1. Findability: How searchable and findable are the DZD CDS items for users and across future versions of DZD CDS?
2. Accessibility: Are the protocols for retrieving the DZD CDS explicit? Do they include well-defined mechanisms to obtain authorization for access to protected data?
3. Interoperability: Are the items in DZD CDS annotated with terms from biomedical ontology terms to support interoperability? Do these annotations include data standards, terminologies and a structured format to enable the automatic extraction of relevant data items across future versions of the DZD CDS?
4. Reusability: Is the DZD CDS presented in a manner concise enough to allow for reuse across all the different future DZD CDS versions and for different studies?

The results of the baseline assessment were used to determine the direction the FAIRification efforts ought to take, facilitate the planning for the various resources that the FAIRification journey would require and motivate the various stakeholders to engage in this journey. This section describes the resources and methods used to FAIRify the DZD CDS.

### The Research Data Alliance FAIR Data Maturity Model

The Research Data Alliance (RDA) was established in 2013 as an international community that aims to address the growing global need for research infrastructure that allows for data sharing across technologies, disciplines, and countries^27^. The RDA working group for a “FAIR data maturity model” was founded in 2019 to develop a common set of core assessment criteria for FAIRness^28^. It established a set of FAIR indicators and related maturity levels. This further led to a set of guidelines and a checklist related to the implementation of the FAIR indicators which are useful for evaluating data FAIRness as shown in the following Table 1^29^. The RDA indicators have been prioritised as follows^30^:

1. Essential: The aspect of this indicator is paramount to achieving data FAIRness. Data FAIRness cannot be achieved without satisfying this indicator.
2. Important: Though the aspect of this indicator is not paramount, satisfying it would significantly increase data FAIRness.
3. Useful: The aspect of this indicator is nice-to-have but is not indispensable.

**Table 1:** FDMM Metrics Indicators.

Indicator ID	Indicator	Priority
RDA-F1-01M	Metadata is identified by a persistent identifier	Essential
RDA-F1-02M	Metadata is identified by a globally unique identifier	Essential
RDA-F2-01M	Rich metadata is provided to allow discovery	Essential
RDA-F3-01M	Metadata includes the identifier for the data	Essential
RDA-F4-01M	Metadata is offered in such a way that it can be harvested and indexed	Essential
RDA-A1-01M	Metadata contains information to enable the user to get access to the data	Important
RDA-A1-02M	Metadata can be accessed manually (i.e. with human intervention)	Essential
RDA-A1-03M	Metadata identifier resolves to a metadata record	Essential
RDA-A1-04M	Metadata is accessed through standardized protocol	Essential
RDA-A1.1-01M	Metadata is accessible through a free access protocol	Essential
RDA-A2-01M	Metadata is guaranteed to remain available after data is no longer available	Essential
RDA-I1-01M	Metadata uses knowledge representation expressed in standardized format	Important
RDA-I1-02M	Metadata uses machine-understandable knowledge representation	Important
RDA-I2-01M	Metadata uses FAIR-compliant vocabularies	Important
RDA-I3-01M	Metadata includes references to other metadata	Important
RDA-I3-02M	Metadata includes references to other data	Useful
RDA-I3-03M	Metadata includes qualified references to other metadata	Important
RDA-I3-04M	Metadata include qualified references to other data	Useful
RDA-R1-01M	Plurality of accurate and relevant attributes are provided to allow reuse	Essential
RDA-R1.1-01M	Metadata includes information about the licence under which the data can be reused	Essential
RDA-R1.1-02M	Metadata refers to a standard reuse licence	Important
RDA-R1.1-03M	Metadata refers to a machine-understandable reuse licence	Important
RDA-R1.2-01M	Metadata includes provenance information according to community-specific standards	Important
RDA-R1.2-02M	Metadata includes provenance information according to a cross-community language	Useful
RDA-R1.3-01M	Metadata complies with a community standard	Essential
RDA-R1.3-02M	Metadata is expressed in compliance with a machine-understandable community standard	Essential

Several tools based on a range of different interpretations of FAIR have been developed to assess data FAIRness in different fields of research^30,31^. The FAIR Data Maturity Model (FDMM) was developed by the RDA as a harmonized set of assessment criteria to assess data FAIRness across the various fields of research^30^. Today it is a community-recognized comprehensive standard for manual FAIR assessment^32,33^. There are two ways of ‘scoring’ the FAIRness of a given resource using these indicators: The first approach scores the progress made per indicator on a five-level scale, while the second assigns a yes/no score to each indicator. The indicators are scored against five levels of compliance based on the degree to which the FAIR principles are implemented as shown in the following Table 2^34^.

**Table 2:** Maturity Levels of FDMM Metrics Indicators.

Score	Compliance Level
0	Not applicable
1	Not considered
2	Under consideration
3	In implementation
4	Fully implemented

In this work the RDA-FDMM approach with five levels of compliance has been used to conduct the FAIR assessment of the DZD CDS. We chose this method because it allowed us to gives the possibility to ‘discard’ the data indicators and focus more on the metadata indicators. It also allowed us to self-assess the evolution of the DZD CDS FAIRifcation journey so as to get a better idea on where to concentrate efforts for a FAIRer outcome. The baseline evaluation has been performed on the former version of the DZD CDS^1,20^. We then used the results of this evaluation to inform our FAIRification journey. Finally, we conducted a second FAIR assessment using the RDA-FDMM to evaluate the FAIRness of the DZD CDS after our FAIRifaction efforts. For purposes of this task, we only scored the RDA-FDMM indicators that addressed the metadata elements which are 26 out of 41. It is important to note that the RDA has indicated that a FAIR evaluation based on the RDA-FDMM should not be conceived as a value judgment but rather as guidance towards improvement of the level of data FAIRness^30^.

## Data Records

**Fig. 1:**
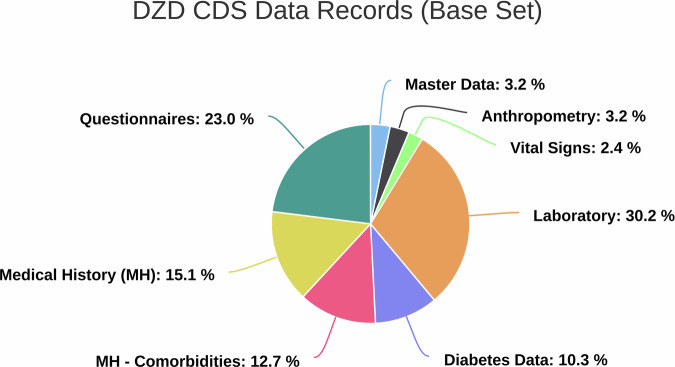
DZD CDS Data Records (Base Set).

The concepts of the DZD CDS are represented in eight modules which include: master data (biodata), anthropometry, vital signs, laboratory, diabetes data, medical history, comorbidities and questionnaires. The modules consist of 126 items. The CDS also has additional optional modules for investigations that are only relevant for special studies. The data types include dates, integers, text (string), floats and booleans. A detailed description of each parameter contributes to the standardization of data collection and ensures that all data are uniformly labeled, defined, and recorded in the same format across all the DZD clinical studies. This is a prerequisite for the comparability of data from one study to another. A representation of the DZD CDS modules and variable proportions is shown in the following Fig. 1.

## Results

This section describes the state of the DZD CDS before FAIRification, the steps taken to FAIRify it, how we implemented these steps and the final FAIRified state of the DZD CDS.

### Findability

#### Former DZD CDS Findability

For purposes of enhanced findability the DZD CDS was published on the DZD website and retrievable through a uniform resource locator (URL)^1,20^. However, this valuable DZD resource was neither registered in a searchable resource nor permanent. More work needed to be done to make the CDS findable in a registry. The metadata contained in the CDS was not standardized and it largely consisted of only the title and the contributors to the data set. There was neither readme nor provenance information provided alongside the data set. Based on this, both the RDA-F1-01M and RDA-F4-01M indicators scored 1 at the FAIR baseline assessment. The RDA-F2-01M scored 2. The RDA-F2-01M indicator scored 3 while the RDA-F1-02M scored 4. The RDA-F3-01M indicator was not applicable in this case.

#### Recommendations based on former state

The need to move the online location of the CDS specification should not require any change in the original URL. Therefore a persistent URL i.e a PURL, is required which then provides a stable, fixed URL that is set to point to content which may be periodically modified^35,36^. Further work is needed to include more metadata in the CDS description to improve its semantic interoperability. This includes enriching the metadata with information about the date on which the data set was completed, contextual information, target audience, keywords that describe the data, license, temporal coverage, spatial coverage, related data sets/resources, file formats used in the data set. The metadata and the data set they describe may be separate files but the persistent identifier (PID) should be explicitly stated in the metadata as indicated in the list of recommended metadata provided. Related readme and provenance information should be provided alongside the data set. The metadata should also include relevant domain specific controlled vocabularies, taxonomies and ontologies. Finally, more work is also needed to register or index the metadata in a searchable resource.

#### Improvements Implemented

**Fig. 2:**
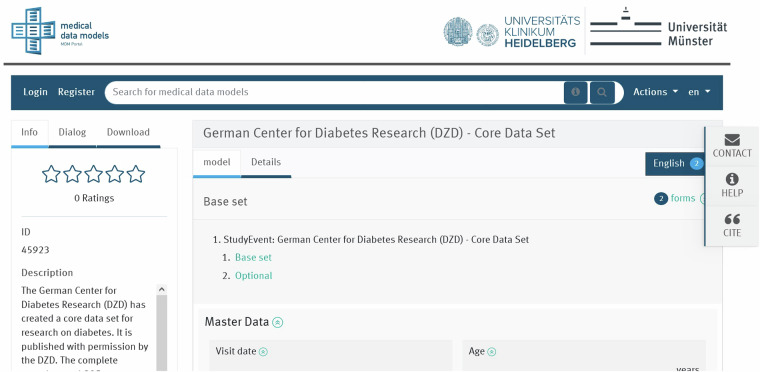
DZD CDS in the MDM portal’s information section with description of the dataset and overview of the model.

**Fig. 3:**
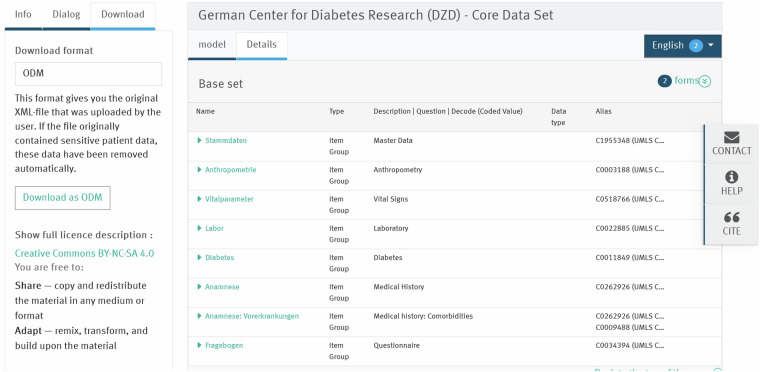
DZD CDS Base Set exploration in the MDM portal. An example for download possibility and details about the modules of the base set.

The DZD CDS was shared at the MDM portal under ID 45923 and Digital Object Identifier (DOI) [10.21961/mdm:45923] as shown in Fig. 2^37,38^. An example for the exploration of the base set of the CDS in the MDM-Portal is found in Fig. 3. The MDM portal is a registered European information infrastructure which provides a multilingual platform for harmonization and exchange of medical data models for medical research for purposes of improving health outcomes^37^. This allows for a PID, versioning and tagging with keywords. It also provides a human-readable description and data type of each data element. Additional metadata and a standard operating procedure (SOP) containing adequate detail to guide research staff through the procedures of the CDS were registered in Zenodo where it was versioned and a DOI was assigned^21,39^. The DOIs on MDM and Zenodo are linked to machine-readable metadata which allows identifiers to stay persistent even after semantic information changes. Codes from the Unified Medical Language System (UMLS) were used to annotate all parameters of the DZD CDS^40^. The addition of metadata in this manner contributes to an overall increased visibility of the DZD CDS. Related readme and provenance information containing the data origin, citations for reused data, description of the data collection, data processing history and version history of the data have been provided alongside the data set. These implementations improved the findability of the DZD CDS. All findability-related metadata indicators scored 4 at the final FAIRness assessment, except RDA-F3-01M which is not applicable in this case.

### Accessibility

#### Former DZD CDS Accessibility

The former version of the DZD CDS was retrievable by ‘clicking on an internet link’ (URL) that is a high-level interface to a low-level protocol that the computer executes to load data in the user’s web browser. The retrieval of the DZD CDS from this URL did not require further mediation by specialised or proprietary tools. This universally implementable protocol also allows for an authentication or authorisation procedure if necessary^41^. The data set which does not contain any personal data was not licensed. Based on this, both the RDA-A1-02M and RDA-A1.1-01M indicators scored 4 while the RDA-A2-01M, RDA-A1-03M and RDA-A1-04M indicators scored 1 at the FAIR baseline assessment. The RDA-A1-01M indicator was not applicable in this case.

#### Recommendations based on former state

Once the DZD CDS is no longer available online it is likely that the URL will become invalid and both humans and machines will no longer be able to access the data set. This creates the need to ensure the persistence of the metadata even after the core data set is no longer available by storing it in a relevant repository. The data set should be licensed.

#### Improvements Implemented

Registering the additional metadata and SOP in the MDM portal and Zenodo allows the metadata to remain persistently accessible even after the CDS is no longer available^21,41^. As the data set does not contain personal data, an open licence was chosen with the help of a tool developed by the Creative Commons that helps the data owner choose an appropriate data license^42^. These implementations increased the accessibility of the DZD CDS. All the accessibility-related metadata indicators scored 4 at the final FAIRness assessment, except RDA-A1-01M which was not applicable in this case.

### Interoperability

#### Former DZD CDS Interoperability

The parameters contained in the DZD CDS have been harmonized across all DZD study centres to define a consistent data set within the DZD clinical studies. In a year-long process all modules and items were adjusted in numerous meetings with speakers, study centers, the Clinical Study Board and the experts for laboratory analyses and health economy. The DZD CDS had references to other related publications^43,44^. However, it was not linked to any other (meta)data that is online resolvable as required according to the FAIR principles. There were also no PIDs as required to link the data set to online available (meta)data. It did not contain any ontologies, controlled vocabularies or thesauri to describe the structure of (meta)data. The qualified references in the DZD CDS were insufficient and the DZD CDS was made available on the website as an MS Excel file. The minimal metadata was neither online resolvable nor linked to any other online resolvable (meta)data. Based on this, both the RDA-I3-01M and RDA-I3-02M indicators scored 3 at the FAIR baseline assessment. All the other interoperability-related metadata indicators scored 1.

#### Recommendations based on former state

It is critical that the machines have knowledge of other system’s data exchange formats^42,45^. This can be facilitated by the implementation of a comprehensive data model that contains ontologies, controlled vocabularies and thesauri to describe the structure and meaning of (meta)data for purposes of semantic enrichment and later retrieval^28,41^. Adding contextual knowledge via qualified references to the data set will facilitate interoperability^41,46^. Qualified references provide descriptions about relationships and associations between (meta)data in a meaningful way^46^. If other data sets are needed to complete the data, or if complementary information is stored in a different data set, these references need to be included in the original data set^41^. Technical formats that give a higher certainty of the machine data readability to spreadsheets are recommended for purposes of reusability and interoperability^31,47^. Proprietary data formats should be avoided to ensure long-term access to the data, independent of a specific software tool^31^.

#### Improvements Implemented

Publishing the DZD CDS at the metadata registry MDM portal allows downloading and exporting the file in most common technical formats such as ODM, PDF, CDA, CSV, FHIR, SQL, SPSS, ADL, R and XLSX. The Clinical Data Interchange Standards Consortium Operational Data Model is the preferred format for download in the MDM portal^48,49^. Some other formats are still under revision. If the CSV format is chosen, the download is split in four categories: One containing the Study OID, the title and description. Two forms (starting with “F1” resp “F2”) containing the Base Set and the Optional Set. The fourth contains the code lists and refers to the forms via the shared identifier in column “OID”. For purposes of standardizing the vocabulary, all the concepts in the CDS were successfully annotated with codes from the UMLS that were provided by the MDM portal^50^. In addition, all parameters in the modules “Vital Signs” and “Laboratory” were annotated with LOINC codes^51^. All parameters from the modules “Master Data”, “Anthropometry”, “Diabetes”, “Medical History”, and “Medical History - Comorbidities” have received an additional annotation with codes according to SNOMED-CT^51,52^. The CDS on the MDM portal has been linked to the complete metadata and SOP which is registered in a searchable resource and is online resolvable^21^.

These efforts resulted in all the interoperability-related metadata indicators scoring 4 at the final FAIRness assessment. To further enhance the interoperability, references to other related data sets such as the CDS of the Medical Informatics Initiative in Germany may be added^22,53^.

### Reusability

#### Former DZD CDS Reusability

As already mentioned, the former version of the DZD CDS lacked in metadata and provenance. Furthermore, it had not been released with a license that stipulates reuse and there was neither information about the linkage of the DZD CDS items nor how they have changed over time. If there were to be future versions of the DZD CDS, users would have to read through the different versions and search for matching data items manually and this is difficult to automate. Domain specific standards which also facilitate reuse were yet to be implemented in this data set. It did however have citations for reused data and publications that informed this data set. For example “Laposata’s Laboratory Medicine: Diagnosis of Disease in the Clinical Laboratory, 2e” or “Diabetes mellitus: Neuer Referenzstandard für HbA1c, Dtsch Arztebl 2009; 106(17): A-805 / B-686 / C-670, Reinauer, Hans; Scherbaum, Werner A.” but the citations were incomplete. Based on this, only the RDA-R1-01M indicator scored 2 while all the other reusability-related metadata indicators scored 1 at the FAIR baseline assessment.

#### Recommendations based on former state

It is easier to reuse data if there is rich metadata attached to the data in a manner that allows to decipher the origin, lineage, usefulness, relevance and how to cite the data in the said context. Therefore, generosity when providing metadata and provenance is highly encouraged. To avoid improper data reuse, explicitness in the elaborations that indicate the conditions under which both humans and machines can reuse the data is also encouraged. It is more likely that other researchers reuse data if the metadata contains domain-specific standards, i.e. (meta)data has the same type, is standardized, follows a community accepted template, contains the same type of data organized in a standardized way, well-established and sustainable file formats and uses a common vocabulary. For example, the Rili-BAEK part A 6.3.2 and ISO 15189 5.8 specify general requirements and a minimum set of information that must be included in a clinical chemistry laboratory report in Germany^54^.

#### Improvements Implemented

Rich metadata including standardized vocabularies was attached to the dataset and made publicly available. The Creative Commons BY-NC-SA 4.0 licence was chosen for this data set, metadata and SOP^42^. This follows the regulations of open definition by the Open Knowledge Foundation^55,56^. Therefore, the material is free to share and adapt for non-commercial use as long as appropriate credit is given and the contributions are distributed under the same licence as the original. Users can also reuse parts of the DZD CDS according to their research needs. The MDM portal has features that show similar datasets for comparability purposes. Users of the MDM portal can comment on the data model which allows user feedback.

As shown in the descriptions of the data elements in the laboratory and diabetes modules, the metadata is in accordance with community accepted standards and recommendations^57,58^. The provenance included for the CDS indicates:

- Origin of data, citations for reused data:
  Where available the citations for reused data were extended, it is found under “Description” at the MDM entry. i.e. for the “Baecke Index leisure index” : ; German version by Wagner P, Singer R: “Ein Fragebogen zur Erfassung der habituellen körperlichen Aktivität verschiedener Bevölkerungsgruppen. Sportwissenschaft” (2003) 33:383-397^43^.
- The workflow description for collecting data:
  The detailed standard operating procedure has been published in Zenodo in PDF^21^. Some of this information is also part of the description in the MDM portal, i.e. for “Type of diabetes”: “According to the practice recommendations of the German Diabetes Association: Definition, classification and diagnosis of diabetes mellitus (Update 11/2020). If “type 3”, please specify subtype in the following question.” or for “Waist Circumference”: “Measured in the middle of the highest point of the iliac crest and the last rib, accurate to the nearest 0.5 cm; see DZD-SOP-DM-002 _Core _data_Set _V1.0”.
- The processing history of data and the version history of data:
  The first version (1.1.0) was published in 2021 as an excel sheet on the DZD website. The revised version was first uploaded to the MDM portal in November 2022 and has since been continuously adapted and further versioned (latest release in February 2024).

**Fig. 4:**
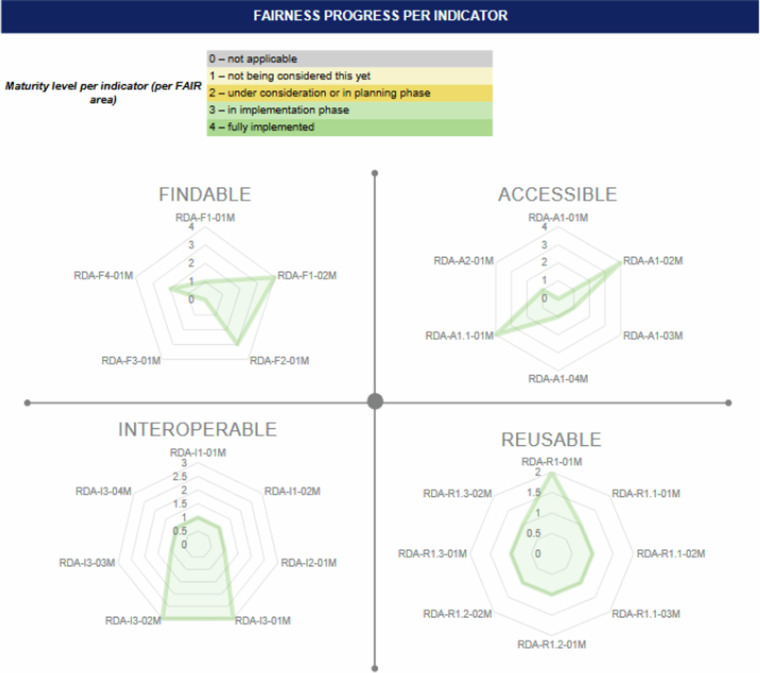
Baseline DZD CDS FAIR assessment results.

**Fig. 5:**
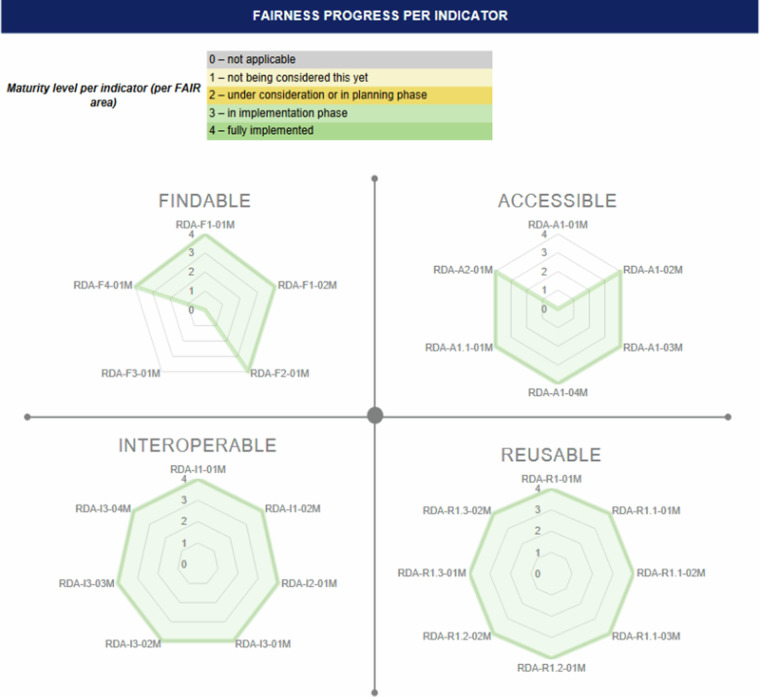
Final DZD CDS FAIR assessment results.

These implementations increased the reusability of the DZD CDS. All the reusability-related metadata indicators scored 4 at the final FAIRness assessment. The following Figs. 4 and 5 show the results of the baseline and final FAIR assessment using the RDA FDMM.

## Discussion

As one step towards comprehensive data stewardship, the DZD data management group prioritised the FAIRification process of the CDS so as to foster its utilisation within the DZD and the wider diabetes research community. This work describes the FAIRification process applied to the DZD CDS. We argue that the enhancement of a CDS enables clinicians to generate and share “better” (FAIR) research data based on the information encoded in the CDS. Specifically, computer-readable data can be shared and exchanged across studies and study sites. The FAIR DZD CDS has been made available to the community in the MDM portal as already shown in Figs. 2 and 3.

In FAIRifying the DZD CDS, it was necessary to experiment with a number of FAIR assessment tools before deciding on which one was the most suitable with regards to the goals, priorities and resources available for the first FAIRification iteration. The decision to use the RDA-FDMM was arrived at after 3 tools were tried, tested and eliminated for various reasons. The SATIFYD was eliminated because although it was extremely easy to use, the experts were not satisfied with the tool’s interpretation of the FAIR data principles^31^. It has since been archived by the developers. The Australian Research Data Commons FAIR Data Self Assessment Tool was eliminated because it did not indicate which FAIR sub-principles are covered by each of the available questions and it does not allow for a separate assessment of data and metadata as was required in this context^59–61^. Although the FAIR Data Self-Assessment-Service for the Human Exposome Assessment Platform was aligned to the goals, priorities and resources available for the first FAIRification iteration, it was eliminated because it provided a similar means of assessment as the RDA-FDMM tool^34^. This process of testing and eliminating FAIR assessment tools was among the time-consuming factors in this work. The RDA-FDMM proved useful in enabling FAIR research data management by explicitly defining the requirements that should be fulfilled to support data FAIRness. The RDA-FDMM also provided for the compliance with these requirements to be evaluated and gaps in compliance to be identified. This further informed our multidisciplinary team that drafted the road map that led to a FAIRer DZD CDS. As the FAIR data principles gain traction, various FAIR assessment tools and frameworks with different focuses and FAIR assessment criteria continue to be developed^62^. These frameworks and tools often show inconsistent and even incomparable results^63,64^. Establishing the criteria that guides the choice of a FAIR assessment tool may prove to be a worthy cause.

The heterogeneity of non-interoperable research data infrastructure in a fragmented landscape makes it challenging to meaningfully exchange data^12^. According to the FAIR data principles, a key part of data interoperability is based on the availability of the data in machine readable formats, the linkage of this data to related (meta)data and the provision of contextual metadata about the data^25^. Semantic annotations as well as mappings to standards and terminologies play a critical role in achieving data interoperability and reusability^44,65^. Prior to our FAIRification efforts, the DZD CDS lacked structured and machine-readable encoding, hindering its potential for broad usability. The data curators thoroughly structured and harmonized the data and mapped the local codes to standardized terminologies in order to provide understandable, valuable and fit-for-purpose data for researchers. The mapping task was not trivial and required domain knowledge as well as thorough understanding of the used standard terminology terms to make sure that the semantic meaning is correctly translated from the local codes to the applied standards. The UMLS was implemented as a formal, accessible, shared code to describe the standardized variables in a machine-readable format. In addition, LOINC codes were provided for all parameters in the laboratory module and SNOMED codes for all others. This is beneficial in a number of ways:

- It contributes to findability by facilitating data intelligibility^66^.
- It allows for data linkage so that the data is openly accessible and shareable semantically which further facilitates reuse^67^.
- It facilitates the data user to identify content in a structured way which facilitates semantic search functionality so that relevant data can be found with a single search query^68^.
- It contributes to the establishment of a well-defined framework to harmoniously describe and structure the DZD CDS which contributes to the increased FAIRness of both the DZD CDS and the related data^69^.
- It lays the groundwork required for data exchange among heterogeneous machines and enables the assessment of the comparability across studies related to the DZD CDS^70^.
- It lays the groundwork required for compatible studies related to the DZD CDS to be combinable in a (semi)automatic way^69,71^.

The ability of the MDM portal to export data in technical formats facilitates the interoperability of the DZD CDS. The implementation of these formats enables data to be loaded directly into heterogeneous software for data analysis, integration and transformation to other formats for purposes of interoperability. The addition of contextual knowledge (PIDs, reference to other data sets/publications) in the form of meaningful links to relevant resources has further enhanced machine-actionability and processing. It may be necessary to provide more details and guidelines such as the specification of conventions for the definition of URIs of common resources to facilitate data interoperability between different data sets that would be relevant for the DZD CDS to be interoperable with. Data validation remains a critical step to ensure that the data generated is usable by researchers and to validate that only valid codes are used to encode the data^72,73^. For purposes of transparent data governance, the data providers developed comprehensive provenance and a README file that detail how the data elements are represented. In all DZD multicentred clinical trials, the direct identifying data (IDAT) are handled spatially and organisationally separated from the medical data (MDAT) to comply with legal, organisational and technical requirements regarding data protection^74^. For this reason the IDAT is not part of the CDS. Record linkage is handled via a pseudonymization service offered by a trusted agency^75^. This influenced our decision to choose an open machine-readable licence^76^. A relevant community standard for diabetes metadata is yet to be implemented in the DZD CDS. Our addition of a plurality of accurate and relevant attributes to the DZD CDS has also increased the reusability of this dataset. Implementing the FAIR principles through independent registry portals like the MDM portal offers both advantages and limitations. These portals are key in making datasets findable and accessible by indexing them with standardized metadata and offering searchable databases, which aids in data standardization and sharing^77^. They enable clinical researchers to merge datasets from various studies, thereby expanding research scope. However, challenges such as gaps in data coverage, inconsistent data quality, sustainability issues, and limited integration with broader data ecosystems diminish their standalone effectiveness^77,78^. This underscores the necessity for integrating registry portals within a more comprehensive data management strategy to enhance their utility in research.

FAIRification has shown to be a worthy investment towards improving the data quality and a FAIR data set positively affects research outcomes^62^. While the FAIR principles are generally known, only a few scientists can explain their meaning or interpretation in detail^79^. Studies show that scientists tend to overestimate the FAIRness of their data^62,64^. Thus, it is imperative to raise awareness among scientists on what it actually means to strive for a “FAIRness” and to support them in the FAIRification process^62,79^.

FAIR data is a journey; not a destination. This work presents our initial FAIRification efforts of the DZD CDS. Yielding consensus on the various matters that tend to arise during the retrospective FAIRification as well as implementing the decisions agreed upon will require a significant investment of resources. We still have a few questions to ponder on as a result of embarking on this journey; What is the frequency of updates required to maintain a FAIR DZD CDS? Is it necessary for all the pertinent stakeholders (data owners, domain experts, FAIR data stewards) to continuously participate in the subsequent FAIRification iterations? Is it a realistic expectation to iteratively provide the resources required for FAIRification cycles? Is it more practical to incrementally set a minimum FAIR score per FAIRification iteration than to aim for the ever elusive 100%?

## Impact of this Work

In this work, we have documented the comprehensive FAIRification journey of the DZD CDS. This work required a deep understanding of the processes by which the CDS had been defined, adapted, and expanded. Conducting this work led us to resonate with the sentiments expressed in previous studies that successful data FAIRification requires collaborative efforts between data stewards, data owners and domain experts^10,80^. The fruit of our collaborative efforts is improved FAIRness of the DZD CDS. We postulate that this result will contribute to enhanced data sharing in diabetes research.

Our FAIRification journey this far has led us to annotate all the concepts in the DZD CDS with UMLS codes which studies show to be a critical step in the implementation of an ontology matching service for querying FAIR data^81^. During the annotation process we established a well-defined framework to describe and structure the DZD CDS in order to facilitate findability and interoperability. We furthermore registered the DZD CDS and the related metadata in the MDM portal and on Zenodo to enable version control and access to current and future versions of the CDS. The MDM portal now houses the DZD CDS in a machine readable format that uses an established and accessible language^37,38^. We expect to see an increase in the reuse of this data set as a result and the fact that we put an open licence on this data set. A key return on investment in the resources employed for this FAIRification journey is the increased certainty of the future data readability. The real world benefit of the applied FAIRification and its limitations remain areas of future research. It would particularly be interesting to see the impact this work has on patient data that has been produced using a FAIRified CDS. We only considered the FDMM metadata indicators in the FAIR assessment of the DZD CDS. This adaptation was necessary to fit the assessment to the context of a CDS. Future work should explore if the methods described in this work are reproducible in a different context.

FAIRification has been described as a gradually incremental process^82^. Our journey so far has helped to increase our understanding of the FAIR concept and informed our decision to employ formal workflows developed for FAIRification for our next FAIRification iteration^80,83^. In aligning more formal workflows to the nature of the DZD CDS and related data sets, it may be necessary to skip the step of data de-identification and pseudonymization because this data set does not contain any information which would comprise the data subjects’ rights regarding privacy issues. Conducting this work led us to resonate with studies that describe the process of retrospectively mapping raw data to the format required for purposes of data transformation and machine-readability as a clerical burden that requires a substantial investment of time and effort^84–87^. It is for this reason that we encourage scientists and data owners to design their scientific projects in a manner that puts into consideration prospective data FAIRification right from the infancy stage. In our case, this largely consists of data standardization and harmonization at the source for purposes of semantic modelling. It has also been recommended that regular FAIR assessments and continuous improvements in FAIR scores should be performed throughout data management^32^.

We hope that this work serves to inform the development of other future FAIR evaluators. It is also anticipated that our FAIRification of the DZD CDS will contribute to its increased uptake among relevant stakeholders both within the DZD the wider diabetes research community and act as a blue print for FAIR core data sets on an international scale.

## Data Availability

An archived record of the former version of the DZD CDS before FAIRification is retrievable in Zenodo at: 10.5281/zenodo.12526690^20^. The FAIRified version of the DZD CDS has been deposited into the MDM portal and it is retrievable at: https://medical-data-models.org/45923?lang=en^38^. The related metadata and SOPs have been deposited into Zenodo and is retrievable at: https://zenodo.org/record/7360000^21^.

## Code availability

No code was used in this work.
